# Unique Functional Profiles in Deafblindness: Evidence From a Comparative Analysis of ICF Core Sets

**DOI:** 10.1177/27536351261481978

**Published:** 2026-08-28

**Authors:** Lorenzo Billiet, Shirley Dumassais, Sarah Granberg, Walter Wittich

**Affiliations:** 1 Ophthalmology, Amsterdam UMC, Location Vrije Universiteit, Amsterdam, The Netherlands; 2 Faculty of Medicine and Health Sciences, Department of Rehabilitation Sciences - Occupational Therapy Research Group, 26656 Ghent University, Ghent, Belgium; 3 École d’optométrie, 5622 Université de Montréal, Montréal, QC, Canada; 4 6233 School of Health Sciences, Örebro University, Sweden

**Keywords:** hearing loss, vision loss, deafblindness, ICF core set

## Abstract

**Objective:**

This study examined whether the functional implications of deafblindness can be understood as the simple sum of vision loss and hearing loss or whether deafblindness constitutes a distinct sensory disability. By comparing the comprehensive ICF Core Sets for vision loss, hearing loss, and deafblindness, the study aimed to determine the extent to which deafblindness reflects unique patterns of functioning beyond additive single sensory difficulties.

**Method:**

A secondary comparative analysis was conducted using all items from the three Core Sets that were classified according to the four ICF components (body functions, body structures, activities and participation, and environmental factors). Items were merged into a single dataset and coded for their presence in each condition. A systematic item-level comparison was performed to identify categories that were common to all three Core Sets, shared by two, or unique to a single sensory condition. No weighting was applied; all items were treated equally. The final output grouped items, reflecting different patterns of overlap and uniqueness.

**Results:**

The Core Sets contained 98 items for vision loss, 118 for hearing loss, and 219 for deafblindness. A total of 435 distinct categories were identified. Of these, 53 items appeared in all three conditions. Overlap showed 27 items shared between vision loss and deafblindness, 34 between hearing loss and deafblindness, and 11 between vision and hearing loss. Unique items were most pronounced in the deafblindness Core Set (n = 105), followed by hearing loss (n = 20) and vision loss (n = 7), indicating a substantial condition-specific functional profile for deafblindness.

**Conclusion:**

The results demonstrate that deafblindness is not merely the additive coexistence of vision and hearing loss but reflects a distinct and more complex functional profile. These findings support the need for condition-specific clinical approaches, policies, and assessment frameworks tailored to the unique demands of deafblindness.

## Introduction

Deafblindness is the combination of vision and hearing difficulties of such severity that the two sensory functions cannot effectively compensate for one another.^1^ As a result, deafblindness constitutes a distinct disability with unique functional, communicative, and participation-related consequences that cannot be understood by considering vision and hearing loss in isolation. A fundamental component of service and research efforts focused on deafblindness is the argument that the combination of vision and hearing difficulties gives rise to a unique type of sensory disability. McInnes^2^ stipulated this early on as a defining feature of deafblindness, specifically where congenital and early-onset sensory difficulties are concerned. He goes into more details in topics such as whether residual vision or hearing abilities are sufficient to compensate for the loss in the other distance sense, and how the failure to do so, and the interaction of the two gives rise to something new – deafblindness. The unique needs of individuals living with deafblindness are intuitively obvious, have been recognized at the policy level,^3^ and the functional implications of deafblindness have more recently been presented in the form of the comprehensive, intermediate and brief Core Sets, following the framework of the World Health Organization’s International Classification of Functioning, Disability and Health.^4-6^

The idea that combining vision and hearing difficulties results in something greater than each impairment alone is immediately recognized by anyone who has worked with individuals with any form or degree of deafblindness.^7^ However, demonstrating this idea that 1 + 1 = 3 or more is not easily accomplished using data. Fuller-Thomson and colleagues,^8^ for example, presented a study on the odds of developing cognitive impairment among older adults, depending on whether they present with a single or combined sensory impairment. Interestingly, the odds ratios indicated that an additive approach of vision (OR = 3.63; 95% CI = 3.59, 3.67) plus hearing (OR = 2.66, 95% CI = 2.64, 2.68),) would predict a combined dual sensory odds ratio of 6.29; however, the actual odds ratio for participants with dual sensory impairment was higher, at 8.16 (95% CI = 8.07, 8.25), demonstrating that a multiplicative effect of dual sensory impairment on the risk of developing cognitive impairment may be present. Abrams^9^ provides an overview of how this effect has been observed on other outcomes, including depression, falls risk, anxiety and even mortality. Such an approach to demonstrate the potential interaction of vision and hearing difficulties is limited to large datasets (in the case of Fuller-Thomson and colleagues, the sample included 5.4 million community-dwelling and institutionalized older adults) and is narrowly focused on one outcome measure at a time. However, even then, such findings do not always replicate,^10^ as this interaction is likely multifactorial and affects multiple functional abilities in parallel.

With the recent completion of the ICF Core Sets for vision loss^11-14^ and deafblindness,^15-22^ and the already existing Core Sets for hearing loss^23-27^ a unique opportunity presents itself for the exploration of whether the implications of deafblindness are simply the addition of those functions prioritized for vision and hearing separately. By comparing the ICF categories that are common to all three Core Sets, those shared by at least two, and those that are unique to each sensory group, we can easily demonstrate the extent to which deafblindness may present with functional impairments above and beyond the combination of its individual components. Therefore, to compare the ICF comprehensive Core Sets for vision loss, hearing loss, and deafblindness, we conducted a systematic item-level comparison across the three conditions.

## Methodology

This study used a secondary analysis approach to explore patterns in ICF Core Set data that are available in the public domain; therefore, no institutional review board ethics approval was required. Each comprehensive Core Set was obtained from its open-access source and included all relevant categories representing functioning and disability for the respective condition.

### Data Preparation and Merging

All items from the three comprehensive Core Sets were compiled into a single dataset. Each item was coded according to its presence in the respective Core Sets (vision loss, hearing loss, deafblindness, see Tables 1-4). This presentation allowed for binary classification (present/not present) across the different conditions. This comparison was performed by systematically checking item occurrence across the three conditions. No weighting or prioritization was applied; all items were treated equally.

**Table 1.** Complete List of Items Included in the Comprehensive Core Sets for Each Sensory Group Within the Body Functions Domain

Domain	Description	Category	Vision loss core set	Hearing loss core set	Deafblindness core set
Body functions	Orientation functions	b114	x		
	Intellectual functions	b117	x	x	x
	Global psychological functions	b122			x
	Temperament and personality	b126	x		x
	Energy and drive functions	b130	x		x
	Energy level	b1300		x	
	Motivation	b1301		x	x
	Sleep functions	b134	x		x
	Attention functions	b140		x	x
	Memory functions	b144	x	x	x
	Psychomotor functions	b147	x		
	Emotional functions	b152	x	x	x
	Perceptual functions	b156	x		x
	Auditory perception	b1560		x	x
	Visual perception	b1561		x	x
	Olfactory perception	b1562			x
	Tactile perception	b1564			x
	Visuospatial perception	b1565			x
	Thought functions	b160	x		x
	Higher-level cognitive functions	b164	x	x	x
	Mental functions of language	b167		x	x
	Reception of spoken language	b16700			x
	Reception of sign language	b16702			x
	Expression of language	b1671			x
	Expression of sign language	b16712			x
	Integrative language functions	b1672			x
	Experience of self and time	b180	x		x
	Experience of self	b1800			x
	Experience of time	b1802			x
	Seeing functions	b210	x	x	x
	Visual acuity functions	b2100			x
	Binocular acuity of distant vision	b21000			x
	Visual field functions	b2101			x
	Quality of vision	b2102			x
	Light sensitivity	b21020			x
	Colour vision	b21021			x
	Contrast sensitivity	b21022			x
	Functions of structures adjoining the eye	b215	x		
	Hearing functions	b230	x		x
	Sound detection	b2300		x	x
	Sound discrimination	b2301		x	x
	Localization of sound source	b2302		x	x
	Lateralization of sound	b2303			x
	Speech discrimination	b2304	x		x
	Vestibular functions	b235	x	x	x
	Vestibular function of position	b2350			x
	Vestibular function of balance	b2351			x
	Vestibular function of determination of movement	b2352			x
	Sensations associated with hearing and vestibular function	b240	x	x	
	Ringing in ears or tinnitus	b2400			x
	Dizziness	b2401			x
	Sensation of falling	b2402			x
	Smell function	b255	x		x
	Proprioceptive function	b260			x
	Touch function	b265			x
	Sensory functions related to temperature and other stimuli	b270			x
	Sensitivity to vibration	b2701			x
	Sensitivity to pressure	b2702			x
	Sensitivity to a noxious stimulus	b2703			x
	Sensation of pain	b280	x	x	
	Voice functions	b310		x	
	Quality of voice	b3101			x
	Articulation functions	b320	x		x
	Fluency and rhythm of speech functions	b330		x	
	Melody of speech	b3303			x
	Fatiguability	b4552			x
	Sensation related to the skin	b840			x

**Table 2.** Complete List of Items Included in the Comprehensive Core Sets for Each Sensory Group Within the Activities and Participation Domain

Domain	Description	Category	Vision loss core set	Hearing loss core set	Deafblindness core set
Activities and participation	Watching	d110	x	x	x
	Listening	d115	x	x	x
	Other purposeful sensing	d120	x		x
	Copying	d130			x
	Acquiring an additional language	d133			x
	Rehearsing	d135			x
	Learning to read	d140		x	x
	Learning to write	d145	x		x
	Acquiring skills	d155	x	x	
	Acquiring basic skills	d1550			x
	Acquiring complex skills	d1551			x
	Focusing attention	d160	x	x	x
	Thinking	d163			x
	Reading	d166	x		x
	Writing	d170	x		x
	Solving problems	d175	x	x	
	Making decisions	d177			x
	Undertaking a single task independently	d2102			x
	Undertaking multiple tasks	d220	x	x	x
	Undertaking multiple tasks independently	d2202			x
	Carrying out daily routine	d230	x		x
	Managing daily routine	d2301			x
	Handling stress and other psychological demands	d240	x	x	x
	Handling stress	d2401			x
	Handling crisis	d2402			x
	Communicating with - receiving - spoken messages	d310	x	x	x
	Communicating with - receiving - nonverbal messages	d315		x	
	Communicating with - receiving - body gestures	d3150			x
	Communicating with - receiving - general signs and symbols	d3151			x
	Communicating with - receiving - formal sign language messages	d320			x
	Communicating with - receiving - written messages	d325	x		x
	Speaking	d330	x	x	x
	Producing nonverbal messages	d335	x		x
	Producing body language	d3350			x
	Producing signs and symbols	d3351			x
	Producing messages in formal sign language	d340			x
	Writing messages	d345			x
	Conversation	d350			x
	Starting a conversation	d3500			x
	Sustaining a conversation	d3501			x
	Conversing with one person	d3503		x	x
	Conversing with many people	d3504		x	x
	Discussion	d355		x	
	Using communication devices and techniques	d360	x	x	x
	Using telecommunication devices	d3600			x
	Using writing machines	d3601			x
	Using communication techniques	d3602			x
	Fine hand use	d440		x	x
	Hand and arm use	d445			x
	Walking	d450	x		x
	Walking on different surfaces	d4502			x
	Walking around obstacles	d4503			x
	Moving around	d455	x		x
	Moving around in different locations	d460	x		
	Moving around within the home	d4600			x
	Moving around within buildings other than home	d4601			x
	Moving around outside the home and other buildings	d4602			x
	Moving around using equipment	d465	x		x
	Using transportation	d470	x	x	x
	Using private motorized transportation	d4701			x
	Using public motorized transportation	d4702			x
	Driving	d475	x	x	x
	Washing oneself	d510	x		
	Caring for body parts	d520	x		x
	Toileting	d530	x		x
	Menstrual care	d5302			x
	Dressing	d540	x		
	Choosing appropriate clothing	d5404			x
	Eating	d550	x		x
	Drinking	d560	x		x
	Looking after one’s health	d570	x		x
	Managing diet and fitness	d5701			x
	Maintaining one’s health	d5702			x
	Acquisition of goods and services	d620	x	x	x
	Preparing meals	d630	x		x
	Doing housework	d640	x		x
	Assisting others	d660	x	x	
	Basic interpersonal interactions	d710	x	x	x
	Social cues in relationships	d7104			x
	Physical contact in relationships	d7105			x
	Complex interpersonal interactions	d720	x	x	x
	Forming relationships	d7200			x
	Interacting according to social rules	d7203			x
	Maintaining social space	d7204			x
	Relating with strangers	d730	x	x	x
	Formal relationships	d740	x	x	x
	Informal social relationships	d750	x	x	x
	Informal relationships with friends	d7500			x
	Informal relationships with peers	d7504			x
	Family relationships	d760	x	x	x
	Parent-child relationships	d7600			x
	Child-parent relationships	d7601			x
	Sibling relationships	d7602			x
	Extended family relationships	d7603			x
	Intimate relationships	d770	x	x	x
	Informal education	d810	x	x	x
	School education	d820	x	x	x
	Vocational training	d825	x	x	x
	Higher education	d830	x	x	x
	Acquiring, keeping and terminating a job	d845	x	x	x
	Remunerative employment	d850	x	x	x
	Non-remunerative employment	d855	x	x	x
	Basic economic transactions	d860	x	x	x
	Economic self-sufficiency	d870		x	
	Personal economic resources	d8700			x
	Community Life	d910	x	x	x
	Recreation and leisure	d920	x	x	x
	Socializing	d9205			x
	Religion and spirituality	d930	x	x	
	Human rights	d940	x	x	x
	Political life and citizenship	d950	x	x	x

**Table 3.** Complete List of Items Included in the Comprehensive Core Sets for Each Sensory Group Within the Environmental Factors Domain

Domain	Description	Category	Vision loss core set	Hearing loss core set	Deafblindness core set
​	Drugs	e1101	​	​	x
​	Products and technology for personal use in daily living	e115	x	x	​
​	Assistive products and technology for personal use in daily living	e1151	​	​	x
​	Products and technology for personal indoor and outdoor mobility and transportation	e120	x	x	​
​	Assistive products and technology for personal indoor and outdoor mobility and transportation	e1201	​	​	x
​	Products and technology for communication	e125	x	x	​
​	General products and technology for communication	e1250	​	​	x
​	Assistive products and technology for communication	e1251	​	​	x
​	Products and technology for education	e130	​	x	​
​	Assistive products and technology for education	e1301	​	​	x
​	Products and technology for employment	e135	x	x	​
​	Products and technology for culture, recreation and sport	e140	​	x	​
​	Products and technology for the practice of religion and spirituality	e145	​	x	​
​	Assistive products and technology for employment	e1351	​	​	x
​	Design, construction and building products and technology of buildings for public use	e150	x	x	x
​	Design, construction and building products and technology of buildings for private use	e155	​	x	x
​	Financial assets	e1650	​	​	x
​	Population density	e2151	​	​	x
​	Climate	e225	​	x	x
​	Light	e240	x	x	x
​	Light intensity	e2400	​	​	x
​	Light quality	e2401	​	​	x
​	Sound	e250	​	​	x
​	Sound intensity	e2500	​	x	x
​	Sound quality	e2501	​	x	x
​	Vibration	e255	​	​	x
​	Immediate family	e310	x	x	x
​	Extended family	e315	x	x	x
​	Friends	e320	x	x	​
​	Acquaintances, peers, colleagues, neighbours and community members	e325	x	x	x
​	People in positions of authority	e330	​	x	x
​	People in subordinate positions	e335	​	x	​
​	Personal care providers and personal assistants	e340	x	x	x
​	Strangers	e345	​	x	​
​	Domesticated animals	e350	x	x	x
​	Health Professionals	e355	x	x	x
​	Other professionals	e360	​	x	x
​	Individual attitudes of immediate family members	e410	​	x	x
​	Individual attitudes of extended family members	e415	​	x	​
​	Individual attitudes of friends	e420	​	x	​
​	Individual attitudes of acquaintances, peers, colleagues, neighbours and community members	e425	​	x	​
​	Individual attitudes of people in positions of authority	e430	​	x	​
​	Individual attitudes of personal care providers and personal assistants	e440	​	x	x
​	Individual attitudes of strangers	e445	​	x	​
​	Individual attitudes of health professionals	e450	​	x	x
​	Individual attitudes of strangers	e455	​	x	​
​	Societal attitudes	e460	​	x	x
​	Social norms, practices and ideologies	e465	​	x	x
​	Communication services, systems and policies	e535	​	x	x
​	Architecture and construction services, systems and policies	e515	​	x	​
​	Housing services, systems and policies	e525	​	x	​
​	Transportation services, systems and policies	e540	x	x	x
​	Civil protection services, systems and policies	e545	​	x	​
​	Legal services, systems and policies	e550	​	x	​
​	Associations and organizational services, systems and policies	e555	​	x	x
​	Media services, systems and policies	e560	x	x	​
​	Media services	e5600	​	​	x
​	Social security services, systems and policies	e570	x	x	​
​	General social support services, systems and policies	e575	x	x	x
​	Health services, systems and policies	e580	x	x	x
​	Education and training services, systems and policies	e585	x	x	​
​	Education and training services	e5850	​	​	x
​	Education and training systems	e5851	​	​	x
​	Labour and employment services, systems and policies	e590	x	x	x
​	Political services, systems and policies	e595	​	​	x

**Table 4.** Complete List of Items Included in the Comprehensive Core Sets for Each Sensory Group Within the Body Structures Domain

Domain	Description	Category	Vision loss core set	Hearing loss core set	Deafblindness core set
Body structures	Structure of brain	s110	​	x	x
	Structure of cranial nerves	s1106	​	​	x
	Structure of eyeball	s220	x	​	x
	Cornea	s2201	x	​	x
	Retina	s2203	x	​	x
	Lens of eyeball	s2204	x	​	x
	Vitreous body	s2205	​	​	x
	Structures around eye	s230	​	​	x
	Eyelid	s2301	x	​	x
	Structure of external ear	s240	​	x	x
	Structure of middle ear	s250	​	x	x
	Structure of inner ear	s260	​	x	​
	Cochlea	s2600	​	​	x
	Structure of head and neck region	s710	x	x	x

### Comparison Procedure

The merged dataset was analyzed to determine patterns of overlap and uniqueness among the three comprehensive Core Sets. This comparison aimed to provide insight into the shared and condition-specific aspects of functioning and disability across difficulties with vision and/or hearing. First, we identified common items, which were categories present in all three Core Sets. These items represent domains of functioning that are universally relevant for individuals with combined or single sensory loss. Next, we examined overlaps focusing on categories shared between two conditions only. These included items present in both deafblindness and hearing loss, items shared by deafblindness and vision loss, and items occurring in both vision loss and hearing loss. These comparisons allowed us to capture areas of partial overlap that may indicate similar functional challenges across two sensory domains. Finally, we identified unique categories appearing exclusively in one Core Set, either deafblindness, hearing loss, or vision loss. Unique items highlight condition-specific aspects of functioning that may require tailored interventions. By classifying all items into these distinct groups, the analysis provided a structured understanding of functioning commonalities and differences across difficulties with vision and/or hearing, which is essential for developing integrated rehabilitation strategies and informing clinical practice.

## Results

### Distribution of Categories Across Sensory Domains

**Figure 1.**
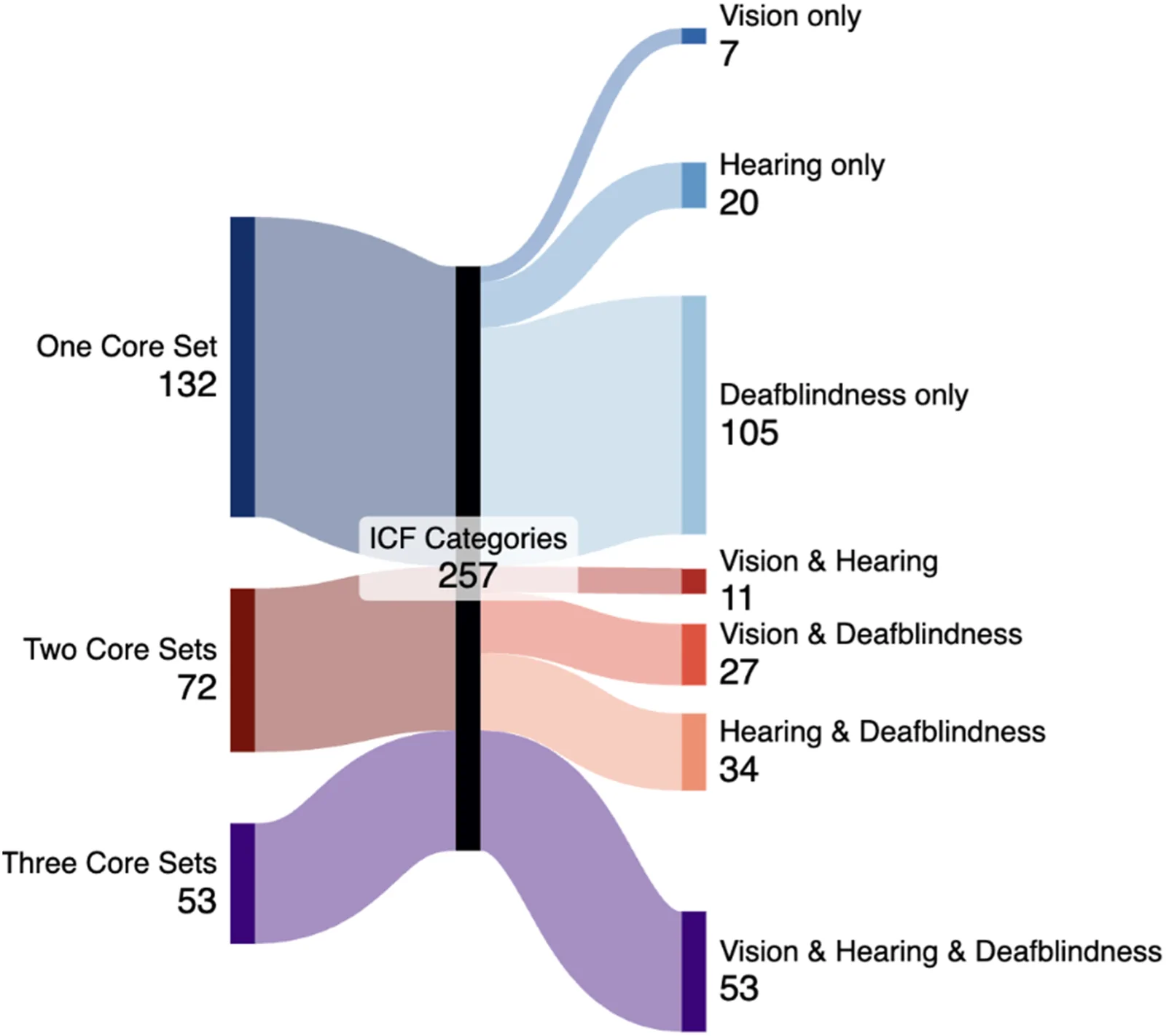
Overlap of items within the comprehensive core sets for hearing loss, deafblindness, and vision loss

The comprehensive Core Set for hearing loss comprised 118 items, for deafblindness 219 items, and for vision loss 98 items. The distribution of items across the ICF components varied by domain. For hearing loss, 5 items were classified under *Body Structures* (s), 22 under *Body Functions* (b), 43 under *Activities and Participation* (d), and 48 under *Environmental Factors* (e). For deafblindness, the respective frequencies were 13 (s), 59 (b), 103 (d), and 44 (e). For vision loss, 6 items were categorized as *Body Structures*, 20 as B*ody Functions*, 52 as *Activities and Participation*, and 20 as *Environmental Factors*. Of the 435 items identified, 53 were common to all three domains, whereas vision loss and deafblindness shared 27 items, while deafblindness and hearing loss had 34 items in common. The overlap between vision loss and hearing loss was limited to 11 items. Condition-specific items included 7 unique to vision loss, 20 unique to hearing loss and 105 unique to deafblindness (available in Appendix A). The overlap among domains is illustrated in Figure 1 and a comprehensive list of the items for each sensory group is available in Tables 1-4.

## Discussion

The comparative analysis of the ICF Core Sets for vision loss, hearing loss, and deafblindness has important implications for service development, knowledge translation and for the implementation of evidence-informed policy and practice. The identification of 105 categories unique to deafblindness, compared with only 20 unique categories for hearing loss and 7 for vision loss, provides strong evidence that deafblindness possesses a substantially broader and qualitatively different functional profile than either single sensory condition. This finding extends beyond a simple increase in the number of affected domains and suggests the emergence of functional demands that are specific to the interaction of vision and hearing difficulties. For knowledge translation, these findings underscore the need to move beyond single-sensory frameworks when communicating evidence, and to explicitly articulate the interactional and compounding functional consequences that emerge only in the presence of deafblindness. The ICF-based comparisons offer a standardized and internationally recognized language through which these complexities can be communicated, facilitating uptake across sectors and jurisdictions, and thereby supporting the advancement of the UN Sustainable Development Goals.^28^ From an implementation perspective, the results challenge policies and practice models that rely on eligibility criteria, assessment tools, and intervention pathways designed for isolated difficulties with vision or hearing. Instead, they support the development of deafblindness-specific assessment, funding, and service models that acknowledge its unique activity limitations, participation restrictions, and environmental support needs. In policy and practice research, recognizing deafblindness as a distinct functional profile strengthens the case for targeted resource allocation, interdisciplinary care models, and outcome measures that are sensitive to deafblindness, thereby increasing the likelihood that research findings translate into meaningful and equitable changes in systems of care.

Deafblindness creates different functional experiences compared to isolated vision or hearing difficulties. The analysis shows that the absence of the natural compensatory relationship between vision and hearing necessitates a reorganization of sensory reliance, cognitive processing, communication strategies, and participation. This is reflected in the prominence of ICF categories related to *touch* (b265), *proprioception* (b260), *vibration* (b2701) and *olfaction* (b255) now become senses that will support access to and carry information. Within *Activities and Participation*, categories related to tactile and sign-based communication are particularly prominent in deafblindness, as they often represent an additional or, in some cases the primary means of communication for individuals with deafblindness. As a result, activities such as *acquiring an additional language* (d133), *communicating with body gestures* (d3150), *signs and symbols* (d3151), and *formal sign language messages* (d320), as well as using *writing machines* (d3601), are central to daily functioning. *Hand and arm use* (d445) is also emphasized, reflecting the physical and tactile demands of communication methods. Among *Environmental Factors*, the central role of assistive technologies is emphasized, not only for general daily living but across specific life domains such as *mobility* (e1201), *education* (e1301) and *employment* (e1351). This highlights that mainstream communication technologies such as cellphones or tablets alone are often insufficient, and that communication generally requires assistive technology such as tactile braille displays or screen readers. This reliance on specialized technologies is closely linked to the unique environmental factor of *financial assets* (e1650), as limited resources may restrict access to technologies and to *education and training services* (e5850). Another environmental factor unique to deafblindness is *drugs* (e1101). Given that deafblindness often involves comorbid health conditions, medication use is more likely and can have a direct impact on daily functioning. The presence of codes unique to deafblindness demonstrate that it cannot be understood as the simple addition of vision and hearing loss. While single sensory loss may converge on shared functional mechanisms, their co-occurrence is multiplicative, not additive, across domains.

Collectively, these findings suggest that the functional profile of deafblindness emerges not simply from the coexistence of vision and hearing difficulties, but from the loss of the compensatory relationship that normally exists between the two senses. Consequently, individuals increasingly rely on tactile, proprioceptive and vibration-based sources of information, while communication, mobility and participation strategies must be reorganized around these alternative channels. The large number of deafblindness-specific categories identified in this analysis provides empirical support for the proposition that deafblindness represents a distinct functional condition rather than the additive combination of two single sensory impairments.

Some ICF categories were present in all three Core Sets because they represent fundamental domains of functioning affected across vision loss, hearing loss and deafblindness. Cognitive and emotional functions such as *intellectual functions* (b117), *memory* (b144), *higher-level thinking* (b164), *attention* (b140, d160) and h*andling stress and other psychological demands* (d240) are central to daily life and are commonly impacted by any sensory difficulty due to the challenges of processing information in limited sensory conditions. Sensory and perception functions including *seeing* (b210), *vestibular functions* (b235), *watching* (d110) and *listening* (d115) are included because all three sensory conditions involve changes in how individuals receive and process environmental input, regardless of the modality affected. The presence of both seeing and hearing functions in all Core Sets underscores the crucial role of senses in compensating for difficulties. Similarly, communication functions such as *receiving spoken messages* (d310), *speaking* (d330), and *using communication devices and techniques* (d360) are consistently relevant because all sensory difficulties influence the ways individuals access information, interact and exchange information with others. Mobility, transportation, economic activities and social participation categories are also common across the three Core Sets reflecting that limitations in sensory input can affect independent navigation, access to goods and services, employment, community involvement and interpersonal relationships, regardless of whether the sensory difficulty is visual, auditory or combined. Finally, political life and advocacy as well as access to essential services such as *transportation* (e540)*, health* (e580)*, employment* (e590) and *social services* (e575) are fundamental determinants of functioning across all sensory difficulties.

### Methodological Considerations and Limitations

This study’s analytical approach has several limitations that warrant consideration. While each ICF development process included in the primary data collection is associated with its own methodological constraints, and is documented in their respective publications, the present discussion focuses specifically on limitations arising from our comparative and integrative secondary analytical strategy. In particular, the act of synthesizing across multiple datasets that were developed by largely independent teams introduces distinct methodological considerations and should be interpreted accordingly. The current study findings are derived from classification data rather than direct input from or validation by individuals with lived experience and professionals. During primary data collection, the original studies incorporated perspectives from professionals and individuals with lived experience through their respective methodologies. Furthermore, our analysis focused on the presence or absence of categories and does not report qualifiers or severity levels, which may limit the nuance of interpretation. In 2009, Jelsma^29^ conducted a literature review on papers utilizing the ICF classification with a specific objective to explore these and other problems encountered regarding the use of the domain categories and of the qualifiers. Three main issues were highlighted in relation to conceptually *overlapping categories, missing categories* and *lack of differentiation of lower order categories (lack of granularity)* (p.4), all of which are considered next. Several of the problems highlighted by Jelsma^29^ are still an issue in the ICF today. Although new categories are added to the classification yearly, or established categories are redescribed, fundamental problems still remain.

#### Conceptually overlapping categories

Methodological and, at times, somewhat arbitrary decisions made during the consensus process are evident in the development of the three Core Sets. When comparing the Core Sets in Tables 1 through 4, twenty categories were identified as unique to hearing loss. However, at a closer look, one can question whether several of these categories can be considered as unique as they may be encompassed within higher-level codes, even if not captured at a more specific level. One example is *voice functions* (b310) where this 2nd level category was used in the Core Sets for hearing loss while the corresponding 3rd level category for *quality of voice* (b3101) was used in the Core Set for deafblindness. Therefore, it is highly questionable whether we should consider these categories as unique to hearing loss or rather a consequence of the consensus conference process.^30,31^ Similarly, the Core Set for vision loss includes the category for *orientation functions* (b114). In contrast, the comprehensive Core Set for deafblindness did not explicitly retain this category but instead captured the concept of orientation indirectly through a range of mobility-related categories. These included *walking* (d450), *walking on different surfaces* (d4502), *walking around obstacles* (d4503), *moving around* (d455), *moving around within the home* (d4600), *moving around within buildings other than home* (d4601), *moving around outside the home and other buildings* (d4602) and *moving around using equipment* (d465). Likewise, the category of *sensation of pain* (b280) was included in both the vision and hearing loss Core Sets. However, it was ultimately excluded from the deafblindness Core Set, despite being initially identified as relevant. This decision was based on the research team’s intent to ensure that included categories reflected functioning directly caused by the combined sensory loss, rather than potential comorbidities.

Furthermore, in line with other studies that have applied linking, the overlap between *attention functions* (b140) and *focusing attention* (d160) was acknowledged in the three Core Sets. The solution to this issue was handled differently in the three Cores Sets considered here. In the mentioned paper by Jelsma,^29^ it was obvious that in several studies both *attention functions* (b140) and *focusing attention* (d160) had been used when linking because the researchers linking the data simply could not distinguish between the two categories. A similar issue was recognized during the different linking procedures in the Core Sets for hearing loss project and the project on deafblindness, and the strategy of “double linking” was applied. When comparing the Core Sets for vision loss, hearing loss and deafblindness it is obvious that the two latter Core Sets adopted this strategy in the linking procedure while the Core Set for vision loss only used focusing attention. This underscores a broader limitation of the ICF. Although the framework is standardized, its application remains influenced by the interpretive decisions of individual research teams. Variations in conceptualization, inclusion criteria, and level of specificity can therefore lead to inconsistencies across Core Sets, even when addressing related or overlapping domains of functioning. On the one hand, the overlap issue is easy to overcome by simply adopting the double linking strategy. On the other hand, this issue also raises concerns about the fundamental principles of the ICF. How can low level activities in the ICF, such as *thinking* (d163) or *focusing attention* (d160), be distinguished from *Body Functions*? One can argue that low level activities require several body functions to cooperate, so from a biological perspective, the distinction makes sense. However, from a clinical and linking perspective, low level activities and body functions might be very difficult to distinguish.

### Missing Categories and Lack of Granularity

It is obvious that the different expert groups involved in the development of these ICF Core Sets have approached the task differently. Compared to prior classifications, the development process of the ICF engaged different groups of experts to assure high validity of ICF categories.^32,33^ It is not clear what kind of guidelines these different groups served under, but it is possible that ambiguities regarding the task could have influenced the development process. In the fields of vision and hearing, specific examples appear. In the Core Sets for vision loss, the category *functions of structures adjoining the eye* (b215) has been used. However, in the Core Sets for hearing loss and deafblindness, corresponding categories in relation to ear structures have not been used. The reason for this matter is that there *are no* corresponding categories when it comes to functions of ear structures. Furthermore, there is an overlap between *hearing functions* (b230) and *auditory perception* (b1560) as several aspects in relation to hearing functions, actually should be viewed as auditory perception functions. The granularity of the categories related to b230 could therefore be questioned for several reasons. These examples of missing categories, overlapping categories and lack of granularity are still a concern when it comes to the actual use of the classification categories. Given the ICF objective to provide a tool for data comparison, concern about both the validity and reliability of certain categories need to be addressed. As with all statistical data, reliability in the data collection procedure is necessary to gain trustworthy and comparable data. Seldom, classification tools are flawless. One must, of course, accept shortcomings in such a comprehensive classification as the ICF. However, clear guidelines on how to handle overlapping categories would benefit everybody who wants to use the categories in the ICF.

## Conclusion

This comparative analysis of the ICF Core Sets for vision loss, hearing loss and deafblindness demonstrates that deafblindness cannot be understood as the simple additive coexistence of two sensory impairments. The large number of categories unique to deafblindness highlights a distinct functional profile characterized by specific communication, participation and environmental support needs. These findings support the development of deafblindness-specific assessment frameworks, rehabilitation approaches and service models, rather than relying on adaptations of vision- or hearing-focused systems. Recognition of deafblindness as a distinct condition is essential for equitable clinical practice, service provision and policy development. In the context of functioning and participation, 1 + 1 does not equal 2.

## Supplemental Material

Supplemental Material - Unique Functional Profiles in Deafblindness: Evidence From a Comparative Analysis of ICF Core Sets Supplemental Material for Unique Functional Profiles in Deafblindness: Evidence From a Comparative Analysis of ICF Core Sets by Lorenzo Billiet, Shirley Dumassais, Sarah Granberg and Walter Wittich in Advances in Rehabilitation Science and Practice.

## Data Availability

The Core Sets for hearing loss are available in the following publication: Granberg, S., Danermark, B., & Gagné, J. P. (2010). The development of ICF core sets for hearing loss. *Perspectives on Audiology*, *6*(1), 20-23. The Core Sets for vision loss are available in the following link: https://osf.io/7xea3/overview. The Core Sets for deafblindness are available in the following link: https://osf.io/a942k/overview.
