# Supplementary material for: Unique Functional Profiles in Deafblindness: Evidence From a Comparative Analysis of ICF Core Sets
Source: Adv Rehabil Sci Pract. 2026 Aug 28;15:27536351261481978. doi: 10.1177/27536351261481978 (PMC13525287; doi:10.1177/27536351261481978)
Supplement: Supplementary file 1 [file sj-pdf-1-rpo-10.1177_27536351261481978.pdf]

Appendix A. Complete list of items unique to the deafblindness Core Set

Domain	Description	Category
**Body functions**	Global psychological functions	b122
	Olfactory perception	b1562
	Tactile perception	b1564
	Visuospatial perception	b1565
	Reception of spoken language	b16700
	Reception of sign language	b16702
	Expression of language	b1671
	Expression of sign language	b16712
	Integrative language functions	b1672
	Experience of self	b1800
	Experience of time	b1802
	Visual acuity functions	b2100
	Binocular acuity of distant vision	b21000
	Visual field functions	b2101
	Quality of vision	b2102
	Light sensitivity	b21020
	Colour vision	b21021
	Contrast sensitivity	b21022
	Lateralization of sound	b2303
	Vestibular function of position	b2350
	Vestibular function of balance	b2351
	Vestibular function of determination of movement	b2352
	Ringing in ears or tinnitus	b2400

	Dizziness	b2401
	Sensation of falling	b2402
	Proprioceptive function	b260
	Touch function	b265
	Sensory functions related to temperature and other stimuli	b270
	Sensitivity to vibration	b2701
	Sensitivity to pressure	b2702
	Sensitivity to a noxious stimulus	b2703
	Quality of voice	b3101
	Melody of speech	b3303
	Fatiguability	b4552
	Sensation related to the skin	b840
**Activities and participation**	Copying	d130
	Acquiring an additional language	d133
	Rehearsing	d135
	Acquiring basic skills	d1550
	Acquiring complex skills	d1551
	Thinking	d163
	Making decisions	d177
	Undertaking a single task independently	d2102
	Undertaking multiple tasks independently	d2202
	Managing daily routine	d2301
	Handling stress	d2401
	Handling crisis	d2402
	Communicating with - receiving - body gestures	d3150
	Communicating with - receiving - general signs and symbols	d3151
	Communicating with - receiving - formal sign language messages	d320

	Producing body language	d3350
	Producing signs and symbols	d3351
	Producing messages in formal sign language	d340
	Writing messages	d345
	Conversation	d350
	Starting a conversation	d3500
	Sustaining a conversation	d3501
	Using telecommunication devices	d3600
	Using writing machines	d3601
	Using communication techniques	d3602
	Hand and arm use	d445
	Walking on different surfaces	d4502
	Walking around obstacles	d4503
	Moving around within the home	d4600
	Moving around within buildings other than home	d4601
	Moving around outside the home and other buildings	d4602
	Using private motorized transportation	d4701
	Using public motorized transportation	d4702
	Menstrual care	d5302
	Choosing appropriate clothing	d5404
	Managing diet and fitness	d5701
	Maintaining one's health	d5702
	Social cues in relationships	d7104
	Physical contact in relationships	d7105
	Forming relationships	d7200
	Interacting according to social rules	d7203
	Maintaining social space	d7204
	Informal relationships with friends	d7500

	Informal relationships with peers	d7504
	Parent-child relationships	d7600
	Child-parent relationships	d7601
	Sibling relationships	d7602
	Extended family relationships	d7603
	Personal economic resources	d8700
	Socializing	d9205
**Environmental factors**	Drugs	e1101
	Assistive products and technology for personal use in daily living	e1151
	Assistive products and technology for personal indoor and outdoor mobility and transportation	e1201
	General products and technology for communication	e1250
	Assistive products and technology for communication	e1251
	Assistive products and technology for education	e1301
	Assistive products and technology for employment	e1351
	Financial assets	e1650
	Population density	e2151
	Light intensity	e2400
	Light quality	e2401
	Sound	e250
	Vibration	e255
	Media services	e5600
	Education and training services	e5850
	Education and training systems	e5851
	Political services, systems and policies	e595
**Body structures**	Structure of cranial nerves	s1106
	Vitreous body	s2205

	Structures around eye	s230
	Cochlea	s2600
